# Laparoscopic Management of Early Primary Abdominal Ectopic Pregnancy: A Retrospective Case Series With an Organ-Preserving Approach

**DOI:** 10.7759/cureus.113477

**Published:** 2026-07-27

**Authors:** Shinichiro Maeda, Kanon Yamashiro, Shigeo Hara, Takuya Aoki

**Affiliations:** 1 Department of Obstetrics and Gynecology, Kobe City Medical Center General Hospital, Kobe, JPN; 2 Department of Obstetrics and Gynecology, Toyooka Hospital, Toyooka, JPN; 3 Department of Pathology, Kobe City Medical Center General Hospital, Kobe, JPN

**Keywords:** early abdominal ectopic pregnancy, laparoscopic surgery, methotrexate, primary peritoneal pregnancy, sigmoid mesocolon

## Abstract

Introduction: Early primary abdominal ectopic pregnancy (EAP) is a rare but potentially life-threatening condition. Preoperative diagnosis is often difficult, and optimal management remains undefined, particularly when the gestational mass is adjacent to vital structures and complete surgical excision carries a risk of organ injury.

Methods: We retrospectively reviewed surgically and pathologically confirmed ectopic pregnancies managed at Kobe City Medical Center General Hospital, a tertiary referral hospital in Kobe, Japan, from January 2015 to December 2025, to identify EAP cases. Diagnosis was based on intraoperative findings and histopathological confirmation of chorionic villi. Clinical characteristics, surgical outcomes, and perioperative serum human chorionic gonadotropin (hCG) trends were analyzed.

Results: Seven cases of EAP were identified among 208 surgically and pathologically confirmed ectopic pregnancies. The mean gestational age at diagnosis was six weeks and one day. Implantation sites were as follows: sigmoid mesocolon (n = 1), pouch of Douglas (n = 3), vesicouterine pouch (n = 1), and posterior uterine wall (n = 2). The mean operative time was 97 minutes, and the mean estimated intraoperative blood loss was 414 mL. All seven cases were managed laparoscopically without major perioperative complications. During laparoscopic surgery, gestational tissue was removed as completely as is safely possible, but intentional conservative excision was performed near the sigmoid mesocolon or bladder to avoid organ injury. Adjunctive methotrexate (MTX) was administered in the sigmoid mesocolon case based on postoperative hCG trends, resulting in complete resolution.

Conclusion: In this small series of early EAP cases, laparoscopic diagnosis and treatment were achieved following timely operative assessment without conversion to laparotomy or major complications. When complete excision poses a risk of organ injury, a stepwise, organ-preserving approach comprising maximal safe excision, postoperative hCG surveillance, and selective MTX may help achieve clinical resolution.

## Introduction

Early primary abdominal ectopic pregnancy (EAP) accounts for approximately 1% of all ectopic pregnancies and carries a maternal mortality rate substantially higher than that of tubal ectopic pregnancy [1-3]. Because clinical presentation is nonspecific and imaging frequently fails to localize the implantation site, preoperative diagnosis of EAP is often difficult. In many cases, EAP is recognized only at surgery performed for a presumed tubal or other ectopic pregnancy [1-4].

Evidence guiding the surgical management of EAP remains limited. In particular, when the implantation site is adjacent to vital organs such as the bowel or mesocolon, aggressive dissection carries a risk of organ injury, and the optimal surgical strategy remains poorly defined [1,2]. Because early diagnosis may allow laparoscopic treatment before extensive placental invasion occurs, accumulating evidence on the clinical presentation and surgical management of EAP is important [1,2].

Against this background, we present a retrospective case series of seven EAPs managed laparoscopically at a single tertiary institution. This study aimed to describe the clinical characteristics, laparoscopic management, and postoperative outcomes of EAP, with particular emphasis on an organ-preserving surgical approach in cases where complete excision may pose a risk of organ injury.

## Materials and methods

We conducted a single-center retrospective descriptive case series at Kobe City Medical Center General Hospital, a tertiary referral hospital in Kobe, Japan, from January 2015 to December 2025. To identify eligible cases, we searched the institutional pathology database for reports containing the terms “ectopic pregnancy” or “heterotopic pregnancy.” All retrieved records were screened without additional sampling, and the corresponding clinical records, operative reports, and surgical videos were reviewed. Because specimens from surgically managed ectopic pregnancies are routinely submitted for histopathological examination at our institution, this approach was expected to capture essentially all surgically managed, histopathologically confirmed cases of EAP during the study period.

EAP was diagnosed based on intraoperative findings, histopathological confirmation of chorionic villi, and retrospective fulfillment of the historical Studdiford criteria as refined by Friedrich and Rankin: (1) normal fallopian tubes and ovaries, (2) no evidence of a uteroperitoneal fistula, (3) implantation exclusively on a peritoneal surface, and (4) no evidence of secondary implantation [5,6]. Cases that did not fulfill these criteria, including tubal, ovarian, or cervical ectopic pregnancies, were excluded.

The first author (SM) extracted the following data from the electronic medical records: maternal age, gravidity and parity, method of conception, gestational age at diagnosis, preoperative imaging findings, implantation site, operative findings, estimated intraoperative blood loss, operative time, adjunctive methotrexate (MTX) use, postoperative hospital stay, perioperative serum human chorionic gonadotropin (hCG) trends, perioperative complications, and subsequent pregnancy outcomes when available. No imputation was performed for unavailable follow-up data; when hCG normalization was not confirmed, the last available hCG value and the reason follow-up ended were reported. Data were summarized descriptively. No statistical testing, adjustment for confounding, or sample size calculation was performed because this was a case series including all eligible cases identified during the study period.

Formal ethics review was deemed unnecessary by the Institutional Review Board Office of Kobe City Medical Center General Hospital under the Ethical Guidelines for Medical and Biological Research Involving Human Subjects in Japan; therefore, no approval number was issued. The study complied with the Declaration of Helsinki, and informed consent was obtained from all patients for treatment and publication of anonymized clinical data.

## Results

During the study period, seven cases of EAP were identified among 208 surgically and pathologically confirmed ectopic pregnancies. The details of the seven cases are summarized in Table 1.

**Table 1:** Summary of seven early primary abdominal ectopic pregnancies (EAPs) G: gravida; P: para; GA: gestational age; w: weeks; d: days; hCG: human chorionic gonadotropin; MRI: magnetic resonance imaging; EP: ectopic pregnancy; IM: intramuscular; Dx: diagnosis; ART: assisted reproductive technology; EBL: estimated blood loss; MTX: methotrexate; POD: postoperative day; ALL: acute lymphoblastic leukemia. †Follow-up was concluded at POD 16, when serum hCG had declined to 1.4 mIU/mL; complete normalization was not formally documented. ‡Follow-up was discontinued at the patient’s request after POD 13, when serum hCG was 21.8 mIU/mL; subsequent normalization was not documented.

Case	Age (years)	G/P/ART	GA at Dx	hCG (mIU/mL)	Preoperative MRI	Operative time (min)	EBL (mL)	Implantation site	Notes	Final hCG follow-up	POD at discharge	Subsequent pregnancy
1	26	G1P0 / ART	6w6d	1,493	Suspected EAP	66	Minimal	Sigmoid mesocolon	Postoperative MTX (single dose IM, POD 13)	Undetectable (POD 42)	4	—
2	29	G4P2 / —	6w2d	1,865	Not performed	67	400	Pouch of Douglas	Suspected endometriosis	Undetectable (POD 19)	2	—
3	34	G2P1 / —	6w1d	1,267	Suspected EAP	60	Minimal	Pouch of Douglas	—	Undetectable (POD 21)	3	—
4	37	G1P0 / ART	5w6d	3,085	Suspected tubal EP	100	100	Vesicouterine pouch	Preoperative MTX at referring hospital; suspected endometriosis	Undetectable (POD 74)	3	Yes (live birth)
5	34	G4P1 / ART	8w1d	616	Suspected EAP or left tubal EP	83	1,000	Posterior uterine wall (left)	Cell salvage reinfusion; ALL in remission (prior chemotherapy)	1.4 mIU/mL (POD 16)†	3	Yes (live birth)
6	34	G1P0 / —	5w1d	14,700	Not performed	114	1,000	Posterior uterine wall (left)	Cell salvage reinfusion; suspected endometriosis	Undetectable (POD 45)	3	—
7	28	G1P0 / —	5w0d	2,061	Not performed	189	400	Pouch of Douglas	—	21.8 mIU/mL (POD 13)‡	4	—

The mean maternal age was 31.7 years (range: 26-37). Three patients had conceived through assisted reproductive technology (ART) (Cases 1, 4, and 5). All seven patients underwent surgical intervention and were diagnosed at an early gestational age (mean: 6w1d, range: 5w0d-8w1d). Review of the clinical records showed that there were no standardized institutional criteria for the timing of operative intervention, the extent of resection, postoperative hCG monitoring, or postoperative MTX use. Postoperative hCG monitoring intervals varied among patients, and postoperative MTX was administered only in Case 1 after serum hCG increased on postoperative day (POD) 13.

Implantation sites were as follows: sigmoid mesocolon (Case 1), pouch of Douglas (Cases 2, 3, and 7), vesicouterine pouch (Case 4), and posterior uterine wall (Cases 5 and 6). The mean operative time was 97 minutes (range: 60-189 minutes); the mean estimated intraoperative blood loss was 414 mL (range: minimal (recorded as 0 mL) to 1,000 mL). All cases were managed laparoscopically without conversion to laparotomy. MTX was administered in two cases, either preoperatively (Case 4) or postoperatively (Case 1). The mean postoperative hospital stay was 3.1 days (range: two to four days). Suspected endometriosis was identified intraoperatively in three cases (Cases 2, 4, and 6). Among those with available follow-up data, subsequent pregnancy was documented in two cases (Cases 4 and 5, both resulting in live births).

Case 1: sigmoid mesocolon implantation (26-year-old, G1P0, ART, 6w6d, hCG 1,493 mIU/mL)

**Figure 1:**
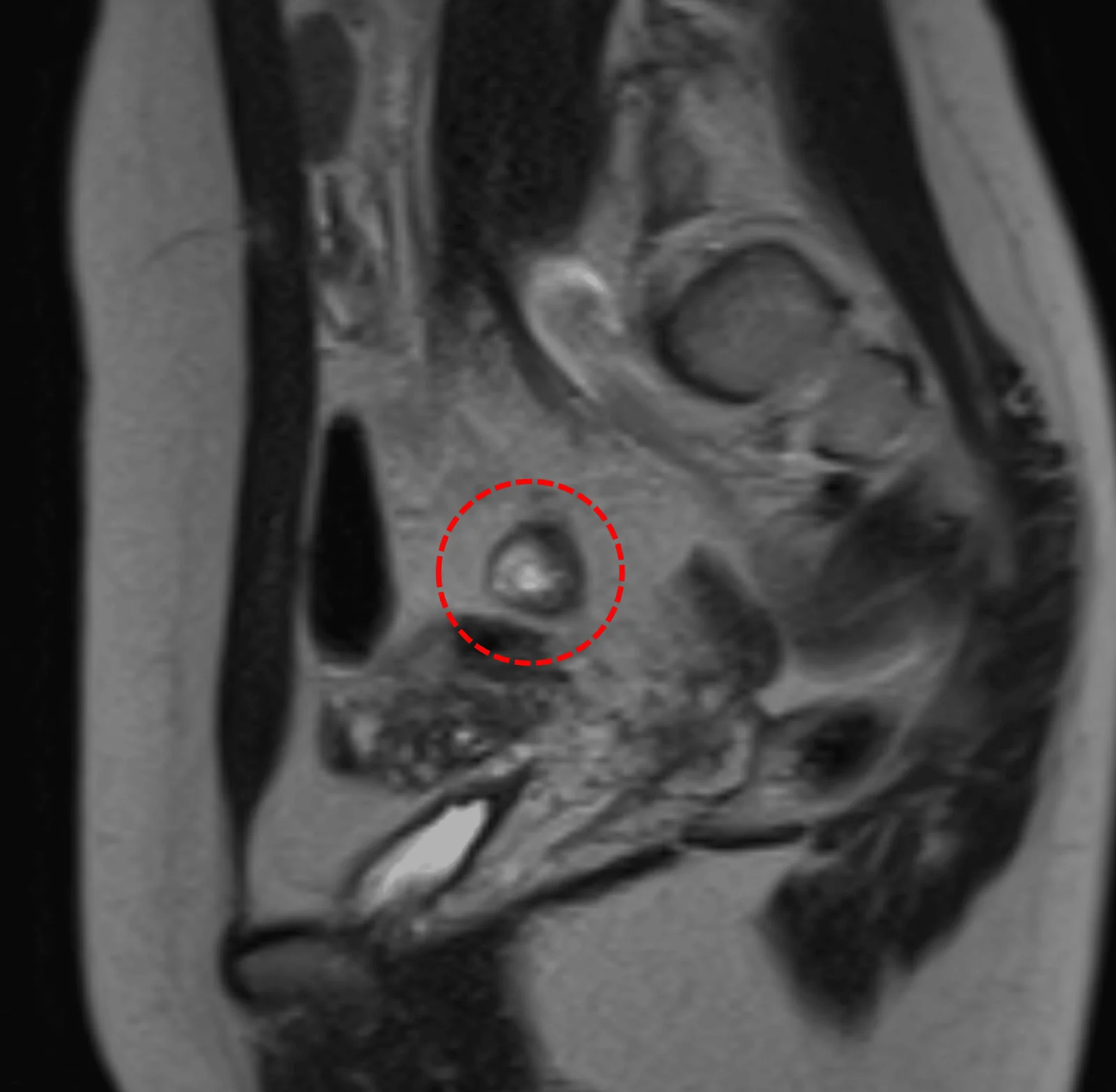
Case 1: Preoperative MRI Preoperative sagittal T2-weighted MRI of Case 1, showing a gestational sac (red dashed circle) in the pelvic cavity, separate from the uterus and adnexa, suggestive of an abdominal ectopic pregnancy.

**Figure 2:**
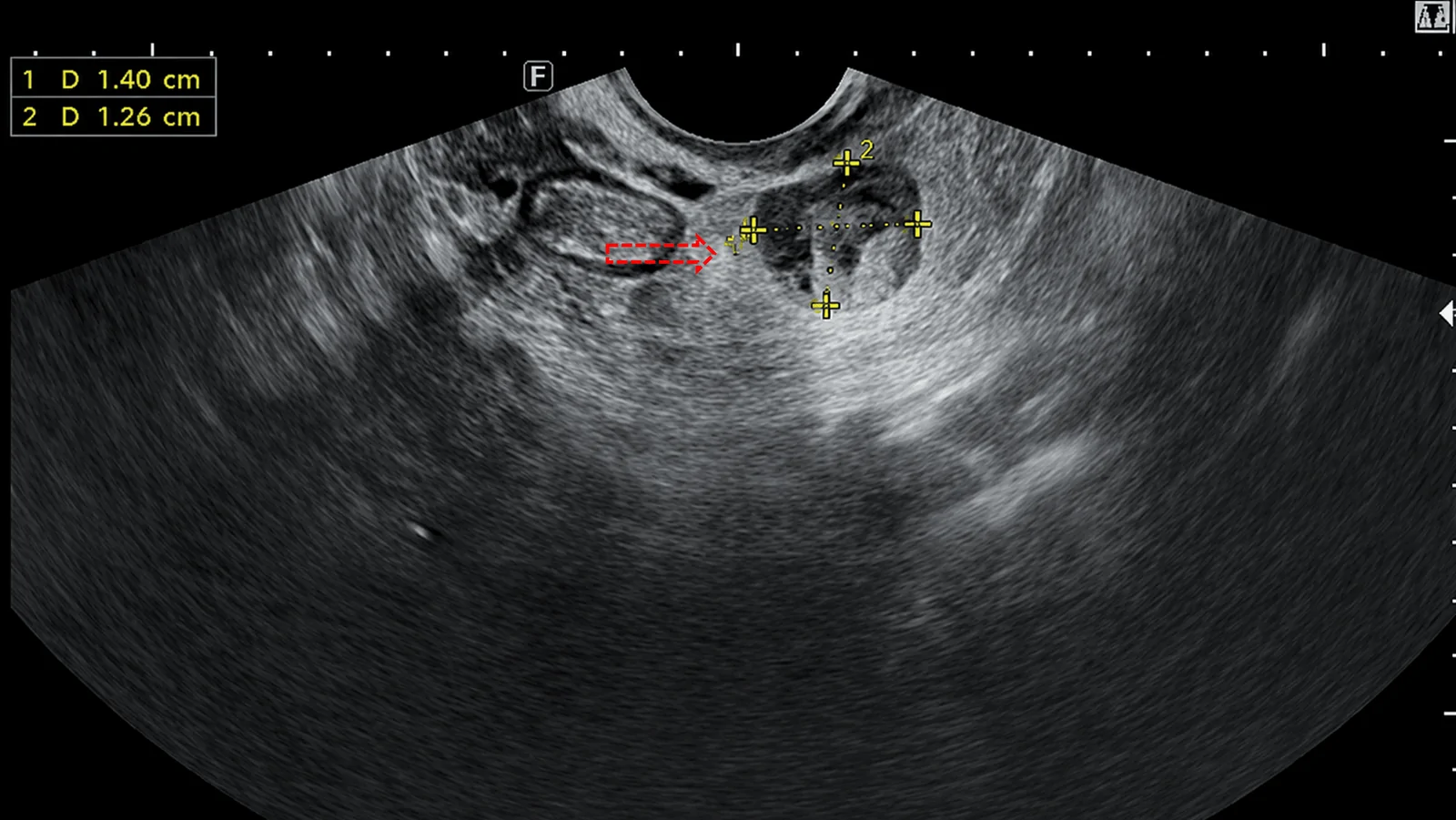
Case 1: transvaginal ultrasound Transvaginal ultrasonography of Case 1, demonstrating a gestational sac-like structure (red arrow), measuring 1.40 × 1.26 cm, in the left pelvic cavity remote from the left fallopian tube.

A 26-year-old nulliparous woman who had conceived through ART was referred to our hospital for evaluation of abdominal pain with free fluid on ultrasound. The hCG level at referral was 760 mIU/mL. Transvaginal ultrasound demonstrated neither an intrauterine nor an adnexal gestational sac. Serial hCG measurements showed a gradual increase to 1,493 mIU/mL, and subsequent MRI suggested implantation within the left pelvic cavity, separate from the adnexa, raising suspicion of EAP (Figure 1). Repeat transvaginal ultrasound identified a suspected gestational sac remote from the left fallopian tube (Figure 2).

**Figure 3:**
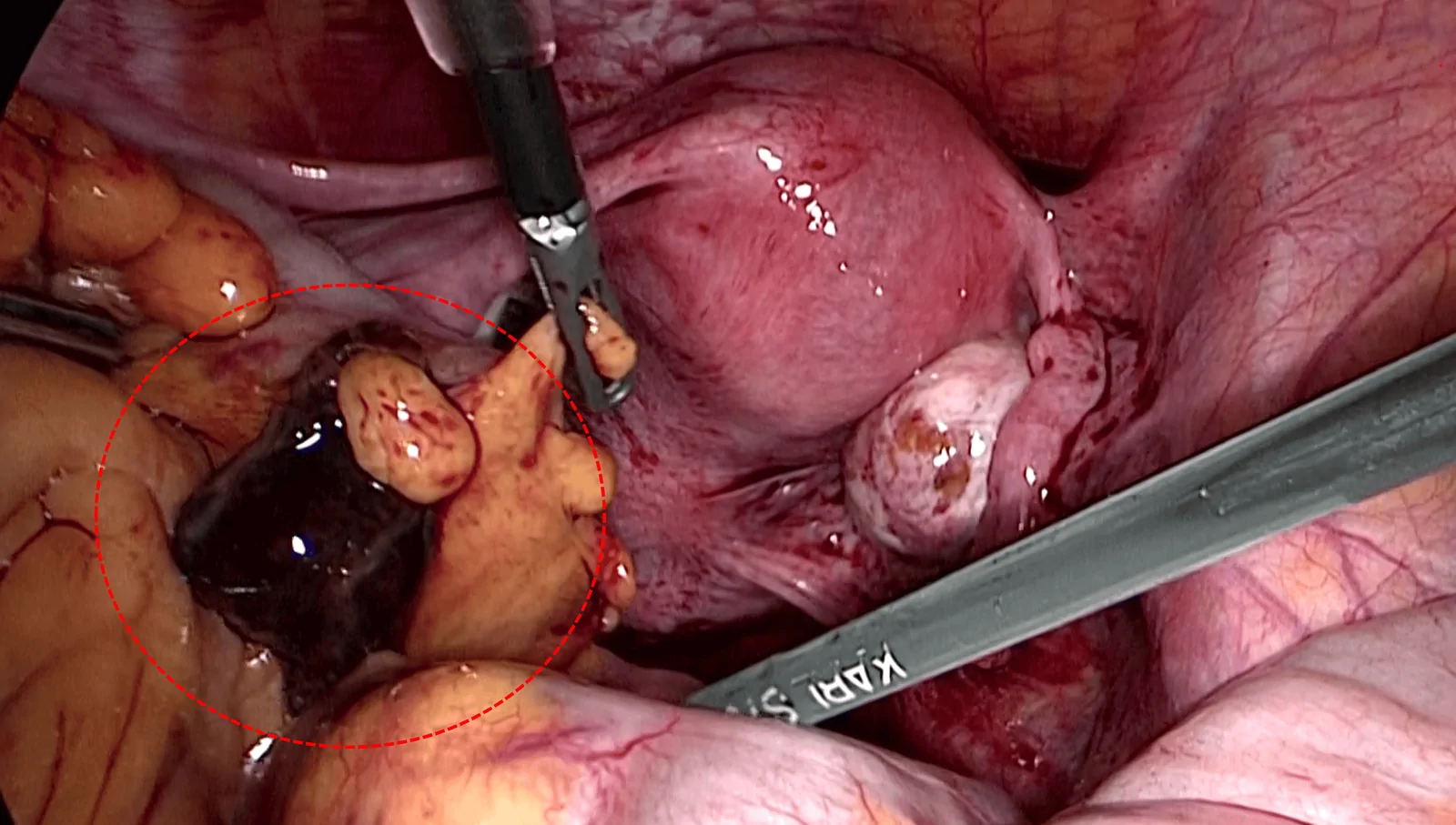
Case 1: sigmoid mesocolon implantation Intraoperative laparoscopic view of Case 1, showing a gestational mass implanted on the sigmoid mesocolon.

**Figure 4:**
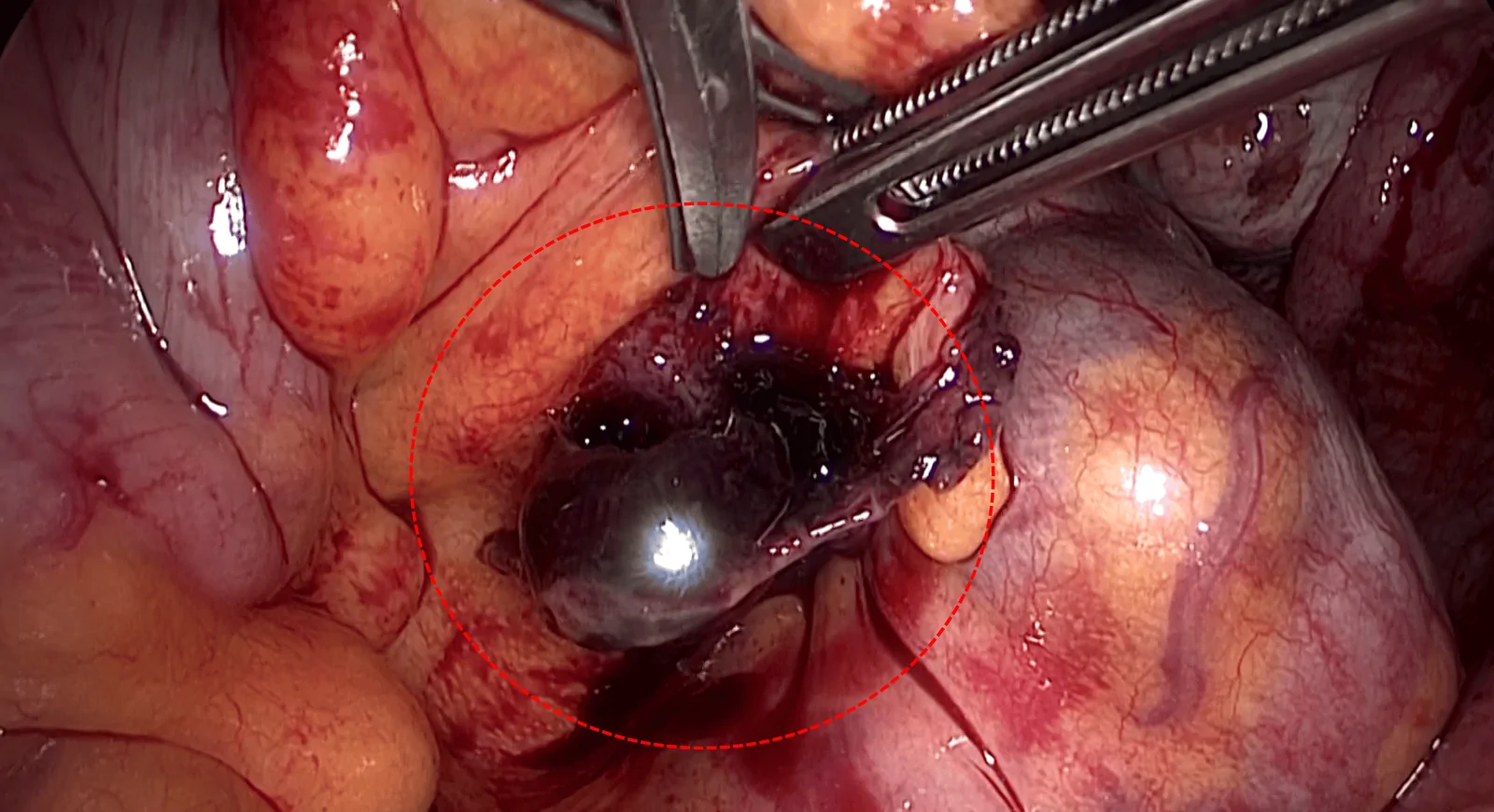
Case 1: close-up view of the implantation site Close-up laparoscopic view of the implantation site in Case 1. Aggressive dissection was deliberately avoided to prevent bowel injury.

We performed diagnostic and operative laparoscopy at six weeks and six days of gestation. Intraoperatively, a gestational mass measuring approximately 2 cm was identified on the sigmoid mesocolon, with no abnormalities of the bilateral fallopian tubes or ovaries (Figures 3, 4).

**Figure 5:**
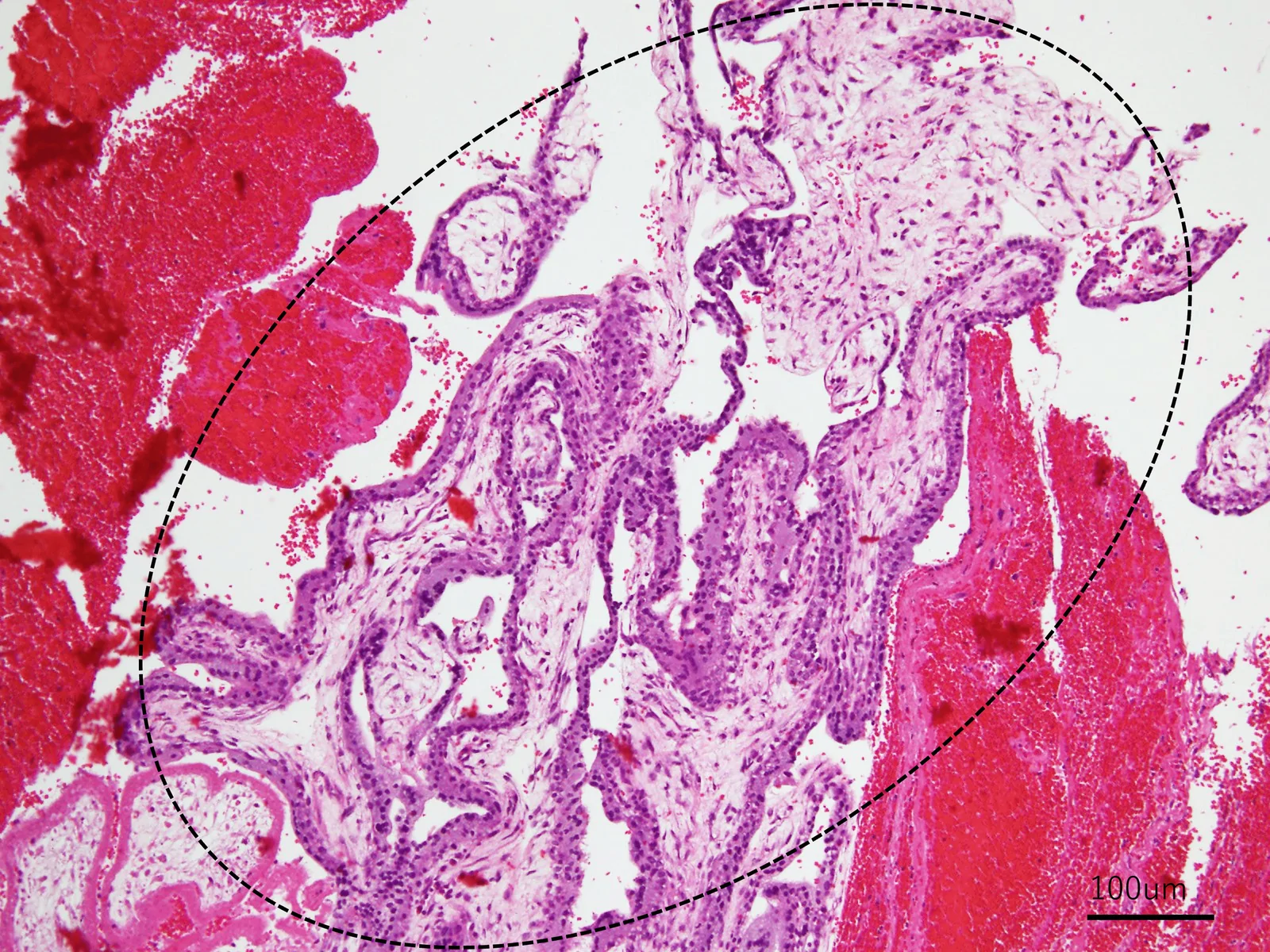
Case 1: histopathological confirmation of chorionic villi Histopathological examination of the resected tissue from Case 1 (hematoxylin and eosin stain, ×100), confirming the presence of chorionic villi consistent with an ectopic pregnancy. Scale bar = 100 µm.

There was no evidence of a uteroperitoneal fistula. These findings, together with histopathological confirmation of chorionic villi, satisfied the traditional criteria for primary abdominal ectopic pregnancy (Figure 5).

Resection of the most visible gestational tissue was performed using grasping and scissor forceps; however, aggressive dissection into the mesocolon vasculature was deliberately avoided to minimize the risk of bowel injury, accepting the possibility of residual trophoblastic tissue. Hemostasis was secured using a topical hemostatic agent. Estimated intraoperative blood loss was minimal, and the operative time was 66 minutes.

Postoperatively, hCG initially declined to 228 mIU/mL on POD 4, but subsequently increased to 263 mIU/mL on POD 13, raising concern for persistent trophoblastic activity. A single dose of adjunctive intramuscular MTX (50 mg/m²) was therefore administered on POD 13. The hCG level subsequently declined to undetectable levels (<1.0 mIU/mL) by POD 42. No complications were encountered.

Case 2: pouch of Douglas (29-year-old, G4P2, 6w2d, hCG 1,865 mIU/mL)

A 29-year-old woman (G4P2) presented to our emergency department with acute abdominal pain at six weeks and two days of gestation. Transvaginal ultrasound demonstrated free pelvic fluid without an intrauterine gestational sac, and hCG was 1,865 mIU/mL. Ruptured ectopic pregnancy was suspected, and emergent laparoscopy revealed a gestational mass implanted in the pouch of Douglas. Laparoscopic excision was performed with an estimated blood loss of 400 mL. The postoperative course was uneventful, and hCG normalized by POD 19.

Case 3: pouch of Douglas (34-year-old, G2P1, 6w1d, hCG 1,267 mIU/mL)

A 34-year-old woman who conceived naturally was referred to our hospital with abdominal pain and vaginal bleeding at six weeks and one day of gestation. Ultrasonography showed a pelvic hematoma without an identifiable gestational sac, and MRI suggested EAP in the pouch of Douglas. Emergent laparoscopy confirmed a hematoma-covered gestational mass in the pouch of Douglas, which was excised laparoscopically with minimal blood loss. hCG normalized by POD 21.

Case 4: vesicouterine pouch (37-year-old, G1P0, ART, 5w6d, hCG 3,085 mIU/mL)

**Figure 6:**
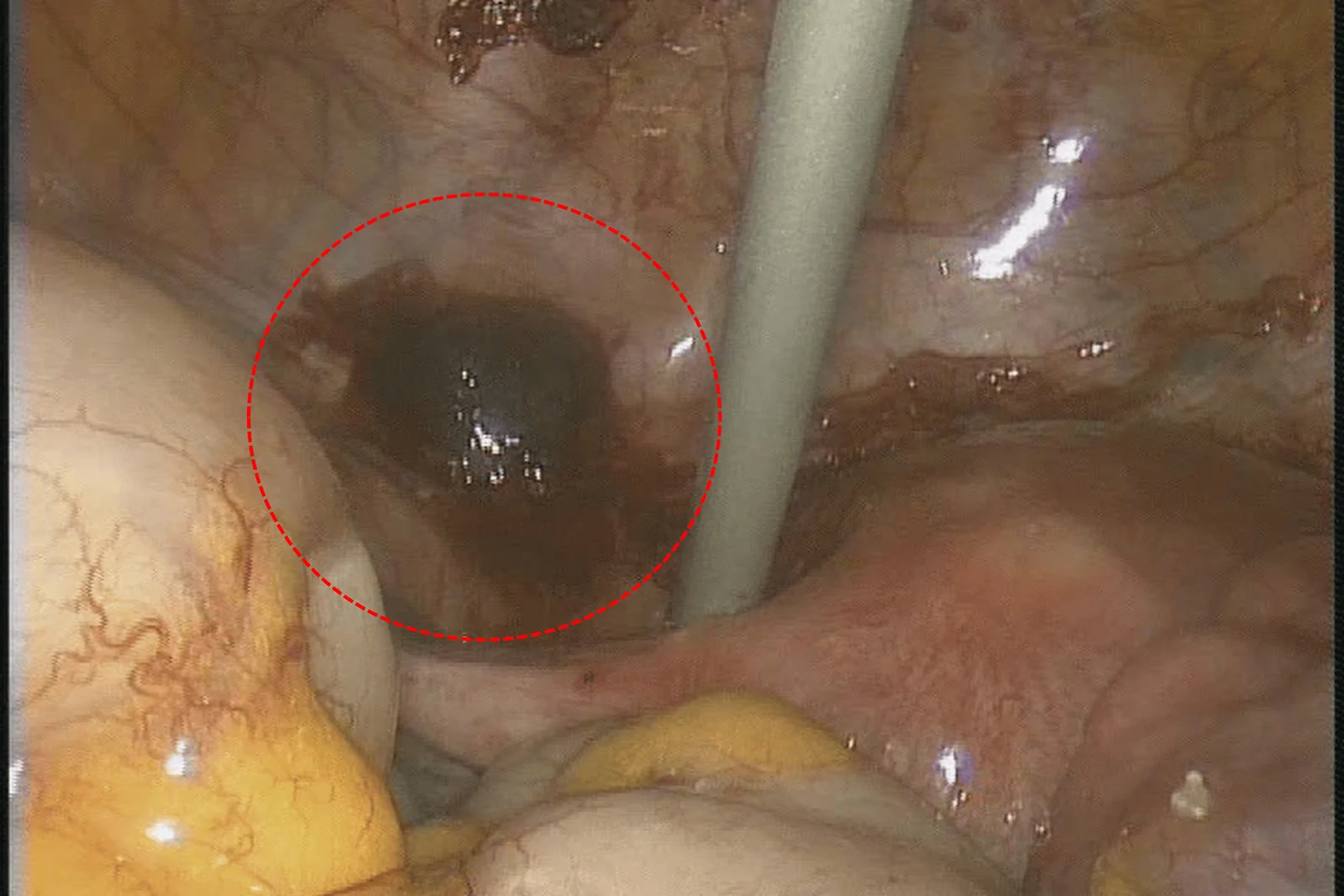
Case 4: vesicouterine pouch implantation Intraoperative laparoscopic view of Case 4, showing a gestational mass implanted on the left vesicouterine pouch.

A 37-year-old nulliparous woman who had conceived through ART was referred to our hospital three days after a single dose of MTX (75 mg) had been administered at a fertility clinic for suspected left tubal pregnancy. hCG had risen from 1,350 mIU/mL before MTX to 3,359 mIU/mL at referral. MRI was interpreted as consistent with left tubal ectopic pregnancy, and no signs of peritoneal implantation were identified. Because hCG remained elevated (3,085 mIU/mL) four days after referral, diagnostic laparoscopy was performed. Intraoperatively, a gestational mass was observed in the left vesicouterine pouch (Figure 6). Because the implantation site was close to the bladder, laparoscopic dissection of the gestational mass was performed with careful attention to avoiding bladder injury. Estimated blood loss was 100 mL. hCG declined to 9.4 mIU/mL by POD 11 and became undetectable by POD 74.

Case 5: posterior uterine wall (34-year-old, G4P1, 8w1d, hCG 616 mIU/mL)

A 34-year-old woman (G4P1) who conceived through ART was referred to our hospital at eight weeks and one day of gestation after a CT scan performed for acute abdominal pain revealed intrapelvic bleeding. Her hCG level at the time of referral was 616 mIU/mL, and MRI suggested EAP or left tubal ectopic pregnancy. Emergent diagnostic laparoscopy revealed a gestational mass implanted on the serosal surface of the left posterior uterine wall, with approximately 1,000 mL of hemoperitoneum. Intraoperative cell salvage with autologous blood reinfusion was performed. The implanted mass was removed laparoscopically, and hCG declined to 1.4 mIU/mL by POD 16, at which point follow-up was concluded.

Case 6: posterior uterine wall (34-year-old, G1P0, 5w1d, hCG 14,700 mIU/mL)

**Figure 7:**
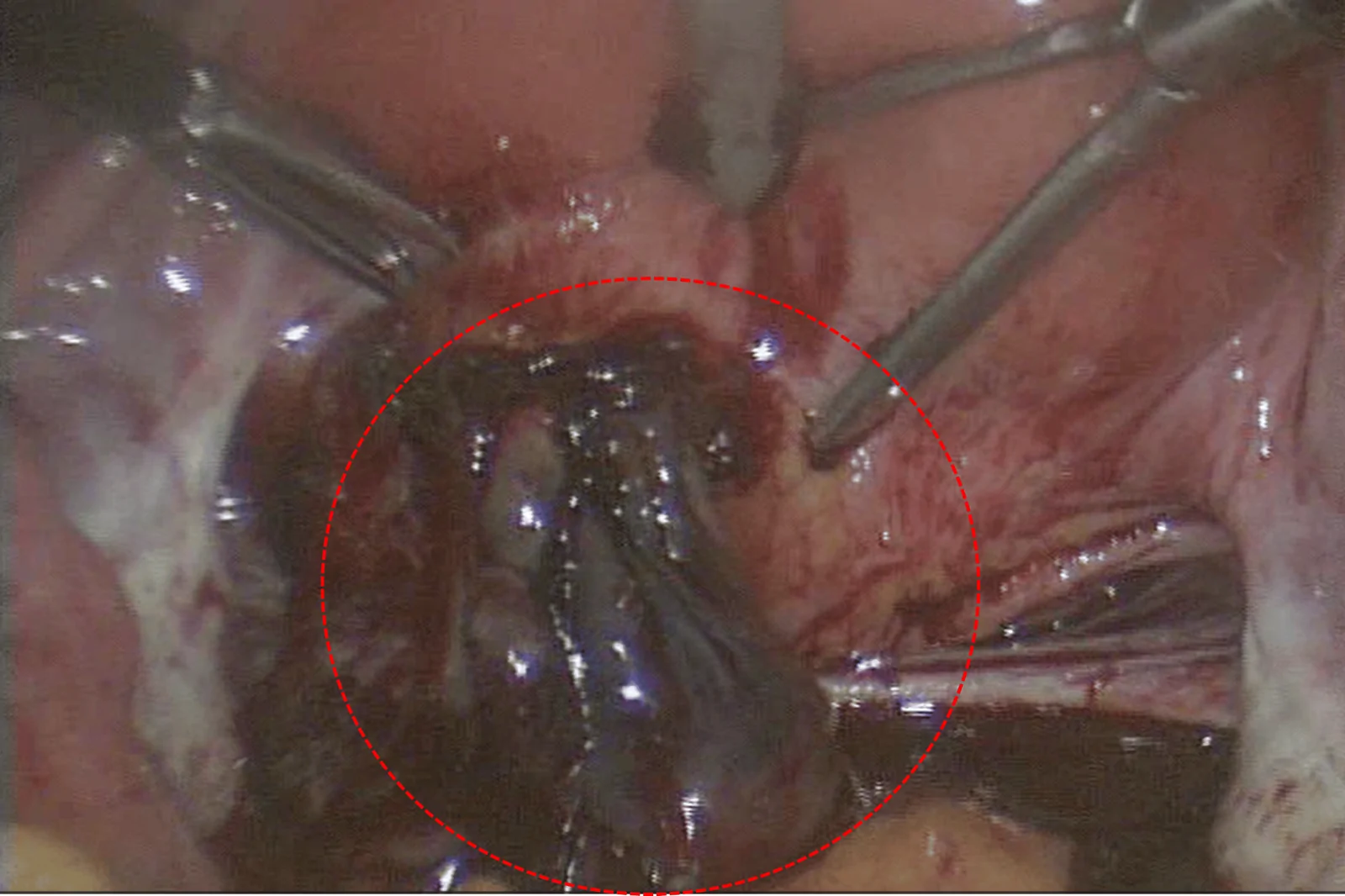
Case 6: ruptured posterior uterine wall serosal implantation Intraoperative laparoscopic view of Case 6, showing a ruptured gestational mass on the left posterior uterine wall serosa with hemoperitoneum. Adjacent adhesions suggested underlying endometriosis.

A 34-year-old nulliparous woman presented with abdominal pain at five weeks and one day of spontaneous gestation. The hCG level was 14,700 mIU/mL. Transvaginal ultrasound showed free pelvic fluid without an intrauterine gestational sac. Because of the clinical urgency, preoperative MRI was not performed. Emergent diagnostic laparoscopy revealed a ruptured gestational mass on the serosal surface of the left posterior uterine wall, with approximately 1,000 mL of hemoperitoneum extending to the hepatic surface (Figure 7). Adjacent adhesions suggested underlying endometriosis. Cell salvage with autologous reinfusion was used. Laparoscopic excision was performed using an ultrasonic coagulation-cutting device and bipolar forceps. hCG normalized by POD 45 without adjunctive MTX.

Case 7: pouch of Douglas (28-year-old, G1P0, 5w0d, hCG 2,061 mIU/mL)

A 28-year-old nulliparous woman with a spontaneous pregnancy presented with abdominal pain at five weeks and zero days of gestation. Transvaginal ultrasound demonstrated no intrauterine gestational sac despite an elevated hCG level of 2,061 mIU/mL. Diagnostic and operative laparoscopy revealed a gestational mass implanted in the pouch of Douglas, which was excised laparoscopically. hCG declined to 21.8 mIU/mL by POD 13, but subsequent normalization was not documented because follow-up was discontinued at the patient’s request.

## Discussion

The main finding of this case series is that early primary abdominal ectopic pregnancies could be diagnosed and treated laparoscopically without conversion to laparotomy or major perioperative complications requiring bowel or bladder repair, reoperation, or postoperative intensive care. This finding is clinically relevant because EAP often presents with nonspecific clinical findings and variable implantation sites, and is therefore difficult to diagnose preoperatively. In this context, laparoscopy may provide both diagnostic confirmation and definitive treatment in appropriately selected early cases.

These outcomes should be interpreted in light of the timely decision to proceed with operative assessment at an early gestational age. Previous reports have also suggested that early gestational age is an important condition for successful minimally invasive management of EAP [1,2,7,8]. In practice, this requires maintaining suspicion for EAP in patients with suspected ectopic pregnancy when the implantation site cannot be clearly localized preoperatively. Preoperative MRI may help localize the implantation site and refine surgical planning [9], but it was not always definitive in our series; favorable outcomes appeared to depend more fundamentally on timely clinical decision-making regarding surgical intervention. Our cases therefore support an approach in which clinicians should actively consider EAP in suspected ectopic pregnancy and proceed to early operative assessment when the diagnosis remains uncertain.

Our series also provides a practical treatment perspective for the laparoscopic management of EAP. Gynecologic surgeons should attempt to remove ectopic gestational tissue as completely as is safely possible; however, complete excision should not be pursued at the expense of organ preservation or hemostatic control. This principle was best illustrated by our sigmoid mesocolon case (Case 1), an uncommon variant of EAP [10-13], in which deeper dissection was intentionally avoided because of concern for bowel and mesenteric vascular injury. This concern is supported by previous reports of bowel- or mesentery-associated abdominal pregnancy in which serious complications or invasive management were documented, including massive gastrointestinal hemorrhage [14], inadvertent bowel injury [15], partial bowel resection to achieve hemostasis [16], emergency laparotomy for ruptured abdominal ectopic pregnancy [17], and laparotomy for advanced gestation with placental attachment to the sigmoid mesentery [18]. In contrast, in Case 1, although postoperative hCG follow-up suggested persistent trophoblastic activity after potentially incomplete excision, complete resolution was achieved with selective adjunctive MTX, without reoperation or complications. Similarly, in Case 4, dissection was intentionally conservative to avoid bladder injury, with acknowledgment of the risk of incomplete trophoblastic clearance; postoperative hCG declined steadily to undetectable levels without additional intervention.

Thus, in light of the clinical goal of safely resolving EAP, a stepwise strategy consisting of maximal safe laparoscopic resection, careful postoperative hCG surveillance, and selective adjunctive MTX when persistent trophoblastic activity is suspected may serve as a practical organ-preserving approach when aggressive surgery could increase the risk of organ injury. Although the available literature is limited, several prior reports support this strategy, describing successful use of postoperative MTX in early abdominal pregnancy when residual trophoblastic tissue was suspected, or complete excision was not feasible [2,19,20]. This strategy may provide a practical treatment framework for EAP, particularly when implantation is adjacent to vital organs or major vessels. Application of this strategy should be individualized according to gestational age, hemodynamic status, implantation site, proximity to vital structures, surgical expertise, and the availability of postoperative hCG surveillance. Familiarity with this safety-oriented strategy is especially important for surgeons because EAP is often diagnosed only intraoperatively rather than before surgery.

This study has several limitations. First, its retrospective single-center design and small sample size (n = 7) limit the generalizability of our findings. Second, the proposed stepwise strategy of maximal safe laparoscopic resection with selective adjunctive MTX was applied on a case-by-case basis rather than according to a predefined protocol, and the indication for MTX was determined by postoperative hCG trends without standardized thresholds. Third, pathology-based case ascertainment may have missed nonsurgical EAP cases, including those treated with MTX alone, although omission of surgically treated cases was unlikely. Fourth, complete serum hCG normalization could not be confirmed in two patients because follow-up ended before hCG became undetectable, resulting in incomplete outcome ascertainment. Fifth, the absence of a comparison group precludes comparative evaluation of the proposed strategy and does not permit conclusions regarding its superiority over alternative management approaches. Finally, because all cases were managed at a tertiary referral institution, our findings may not be directly applicable to all clinical settings. Given the rarity of EAP, prospective studies may be difficult to conduct; accumulation of additional cases through multicenter collaboration or registry-based data collection will be needed to validate these observations.

## Conclusions

Laparoscopic surgery appears to be a feasible treatment option for selected cases of primary abdominal ectopic pregnancy when operative assessment is undertaken at an early gestational age. In cases involving implantation near vital structures such as the bowel, mesocolon, or bladder, complete excision should be balanced against the risk of organ injury. Our experience supports a safety-oriented stepwise strategy consisting of maximal safe resection, close postoperative hCG surveillance, and selective adjunctive MTX when persistent trophoblastic activity is suspected. This organ-preserving approach may help gynecologic surgeons achieve clinical resolution while minimizing the need for laparotomy or organ resection.
